# Dyslexic Adults Can Learn from Repeated Stimulus Presentation but Have Difficulties in Excluding External Noise

**DOI:** 10.1371/journal.pone.0027893

**Published:** 2011-11-23

**Authors:** Rachel L. Beattie, Zhong-Lin Lu, Franklin R. Manis

**Affiliations:** 1 Department of Psychology, University of Southern California, Los Angeles, California, United States of America; 2 Department of Psychology, The Ohio State University, Columbus, Ohio, United States of America; University of Leicester, United Kingdom

## Abstract

We examined whether the characteristic impairments of dyslexia are due to a deficit in excluding external noise or a deficit in taking advantage of repeated stimulus presentation. We compared non-impaired adults and adults with poor reading performance on a visual letter detection task that varied two aspects: the presence or absence of background visual noise, and a small or large stimulus set. There was no interaction between group and stimulus set size, indicating that the poor readers took advantage of repeated stimulus presentation as well as the non-impaired readers. The poor readers had higher thresholds than non-impaired readers in the presence of high external noise, but not in the absence of external noise. The results support the hypothesis that an external noise exclusion deficit, not a perceptual anchoring deficit, impairs reading for adults.

## Introduction

Developmental dyslexia is a disorder of reading acquisition not caused by obvious neurological or sensory impairments. Approximately 5–17.5% of the population experience difficulties in learning how to read [1]. Individuals with developmental dyslexia typically have an inability to process accurately the sound structure, or phonology, of words, which has been considered the core deficit in the manifestation of reading problems [2]. Considerable effort has been expended investigating whether phonological processing and reading problems are a product of more basic deficits in auditory or visual perception. A puzzling finding is that only a minority of dyslexic readers show consistent auditory or visual deficits [3]–[4].

One attempt to account for the observation of reading and phonological processing problems, coupled with occasional difficulties on visual and auditory processing tasks, is the *external noise exclusion hypothesis*. The central idea is that the behavioral manifestations of dyslexia are at least partly due to a difficulty in excluding irrelevant, background information, or noise [5]. When we attend to specific elements in our environment, we are also bombarded with a large amount of irrelevant visual and auditory information. Non-impaired readers filter out this noise so that the target information, or signal, can be processed and then categorized or represented. However, individuals with dyslexia have a particular difficulty in perceiving visual or auditory signals in the presence of distracting information. According to the external noise exclusion hypothesis, the inability to effectively filter out irrelevant information leads to poor categorization of letters and sounds, which ultimately manifests in reading problems. While some studies have found evidence of a direct link between noise exclusion and reading [6]–[8] other studies have only found an indirect link to reading problems through general language abilities [9]. Thus, the exact nature of the noise exclusion deficit is unknown.

Alternatively, the *perceptual anchor hypothesis* explains that dyslexia is underpinned by an inability to learn and construct a perceptual representation from repeated presentations of the same stimulus [10]–[11]. When repeatedly presented with a small set of stimuli, such as when learning by rote, non-impaired readers can automatically form an internal reference, or a psychological anchor [12] to this limited set of items. By forming an anchor, non-impaired readers are able to make faster and more accurate perceptual judgments. On the other hand, if non-impaired readers are presented with a large set of stimuli that varies from trial to trial, they are unable to form an anchor and the task becomes more effortful. Unlike typical readers, children and adults with dyslexia did not show the anchor effect - i.e. did not show a benefit when processing a small, limited set of stimuli. Rather, they performed equally well whether the task had a small or a large stimulus set [13]–[14]. Similar to some of the studies supporting the external noise exclusion hypothesis, the perceptual anchoring deficit appears only to be present in individuals with dyslexia and additional language difficulties.

Although the predictions of these hypotheses have not been directly compared, it has been proposed that dyslexic individuals' inability to exclude external noise was in fact, an anchoring deficit [10]. In tasks used to assess the noise exclusion hypothesis, a small set of visual or auditory stimuli was used rather than a larger set of stimuli. According to the perceptual anchor hypothesis, the use of a limited display set provides a target that non-impaired readers can use to form an internal reference. In contrast, dyslexic readers' failure on the noise exclusion task could in actuality be attributed to an inability to take advantage of repeated stimulus presentation. In order to test whether perceptual anchoring can account for differences in external noise exclusion, the present study assessed low level perceptual processing with both small and large stimulus sets, and in the presence and absence of external noise.

To directly compare the predictions of the External Noise Exclusion and Perceptual Anchor hypotheses, the current study recruited college undergraduates with and without dyslexia, based on tests of word identification and phonological decoding. A visual letter detection task was given that varied two aspects of presentation: the presence or absence of background external noise, and the use of a small or large stimulus set. In the condition with the small stimulus set, the letter identity and placement was held constant. For the condition with the large stimulus set, the identity and location of the letter varied from trial to trial. The external noise exclusion hypothesis predicts that the performance of the dyslexic group on the letter detection task would be significantly worse only when background noise was present. The perceptual anchor hypothesis predicts larger group differences in the small stimulus set conditions (with and without noise) than in the large stimulus set conditions. The study design also tests the hypothesis that the effects of noise and stimulus set size might be additive, or might interact.

## Methods

### 1.1 Ethics Statement

The research participants in this study gave written informed consent and were treated in accordance with ethical standards. The Institutional Review Board at the University of Southern California approved this study.

### 1.2 Participants

Thirty-seven undergraduate students (mean age = 20.43, 22 females) participated in this study. Participants in this study all met the following criteria: 1) an average to above average score on the Spatial Relations subtest of the Woodcock Johnson-III (WJ-III) [15]; 2) normal or corrected-to-normal vision and hearing; and 3) no additional behavioural or emotional disorders. These criteria were established to eliminate other plausible alternatives in task performance. To qualify for the non-impaired readers group, participants had to achieve scores at or above the 40^th^ percentile on both the Word Attack and Word Identification subtests of the WJ-III. The participants who qualified for the poor readers group achieved scores below the 25^th^ percentile on either Word Attack or Word Identification. By using this criteria, the non-impaired readers were average to above-average readers whereas the poor readers were deficient on sight word reading and/or word decoding. Twenty-one of the students qualified for the average to above average readers group and sixteen undergraduate students met the criteria for the poor readers group.

### 1.3 Reading, Phonological Awareness, and Language Measures

In addition to the Word Identification and Word Attack tests, the subjects' reading fluency was assessed with the Gray Oral Reading Test (GORT) [16] and the Test of Word Reading Efficiency (TOWRE) [17] and their reading comprehension was measured using the Nelson Denny Reading Test, a passage reading measure normed on college students [18]. The participants' ability to read exception words, that is, words that do not follow the letter to sound correspondences in English, was also measured [19]. Additionally, the Spelling subtest of the Woodcock-Johnsoon III was used to assess the participants' spelling. The participants' phonological processing skill was assessed with the Rapid Picture Naming and Auditory Working Memory subtests from the Woodcock-Johnson III [15] and the Phoneme Elision subtest from the Comprehensive Test of Phonological Processing [20]. The participants' language abilities were measured using the Verbal Comprehension test and their non-verbal ability was measured using the Spatial Relations test, both from the Woodcock-Johnson III [15].

### 1.4 Letter Detection Task

The letter detection task was programmed using Matlab 7.4, with the Psychophysics toolbox extension, Version Three (PTB – 3) [21]–[22]. The experiment was conducted on a PC computer with a monitor that had a 640×480 pixel resolution and a refresh rate of 75 Hz. The screen and stimulus luminance were determined by measuring a Tektronicx J15 photometer. The mean background luminance was 16 cd/m^2^. The participants were seated 210 centimeters or 82.7 inches away from the computer screen and were given the opportunity to fully adapt to the light levels in the test room.

The letter detection task used a two-alternative forced-choice (2AFC) design. A fixation cross appeared at the center of the screen for 250 ms, and remained on for the duration of the trial. Participants were shown two simultaneous stimulus regions on both sides of the fixation cross for 200 ms. Each stimulus region subtended a 1.65° by 1.58° visual angle. The space between stimulus regions was 3° of visual angle and thus the entire display subtended a 6.3° by 1.58° visual angle. In this 2AFC task, only one of the stimulus regions contained a letter and the participant had to indicate which region that was by pressing either “/” for right side or “z” for the left side. The target letter subtended a 0.40° by 0.40° visual angle, which is comparable to the size of letters in typical reading situations (3–4 letters per 1° visual angle) [23]. A simple computer “beep” was played when the participant answered correctly and a discordant combination of chords (G and Ab) was presented when the subject answered incorrectly. The outcome measure was the contrast threshold for letter detection.

There were two main experimental manipulations: the stimulus set size (large vs small) and the absence or presence of external noise (with noise vs. without noise). For the small stimulus set size conditions, both the letter identity, “X”, and placement in the center of the box were held constant. In the large stimulus set size conditions, the letter identity was randomly selected from a set of fifty-two letters (all letters of the alphabet, uppercase and lowercase) and varied from trial to trial. The letter placement within the box also varied from trial-to-trial in the large stimulus set size condition.

For the second experimental manipulation, the letters were either presented in a condition without external noise or in a condition with noise. Checkerboards composed of 2×2 pixel areas, each subtending a 0.03° by 0.03° visual angle, were used to create the background of the stimulus regions. In both the trials with and without noise, the noise elements and the letter, or signal, elements occupied 50% of the checkerboard pattern. In the trials without external noise, the background of both stimulus regions matched the grey background of the rest of the display whereas in the trials with noise, a noise checkerboard was present in both stimulus regions. The contrast of each pixel patch was sampled from a Gaussian distribution with a mean of 0 and standard deviation of 0 (in the condition without noise) and 0.33 in the condition with noise.

For this study, we used an adaptive procedure that converges to any specified accuracy level to control task accuracy or difficulty. The accelerated stochastic approximation method [24]–[25] converges to a target performance level φ. In the first two trials, the sequence is based on the stochastic approximation procedure [26] and given by:
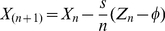
(1)where n is the trial number, X_n_ is the feature value (e.g., stimulus contrast) in trial, Z_n_ is the response accuracy in trial (0 if incorrect or 1 if correct), X_n+1_ is the feature value for the next trial, and s is the pre-chosen step size at the beginning of the trial sequence. From the third trial on, the sequence was “accelerated”:
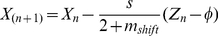
(2)where m_shift_ is the number of shifts in response category (switches from consecutive correct responses to incorrect responses and vice verse). A reasonable stopping criterion would be a lower limit for the step size and an obvious final estimate is the last tested level. In an influential review of adaptive psychophysical procedures, the accelerated stochastic approximation procedure was recommended as the best available procedure for measuring thresholds [27]. In this study, we used a fixed number of trials in order to equate the amount of practice. All of the experimental and practice conditions were equally difficult because we measured contrast thresholds at a fixed accuracy level.

The letter detection task had a total of 260 trials. The experimental conditions were preceded by two practice conditions, the small stimulus set size condition with and without background noise. Each practice condition started at a high contrast level and had thirty trials. The end values of the practice conditions with and without noise were used as the starting value for the experimental conditions with and without noise, respectively. Each of the experimental conditions had fifty trials each and the order of the conditions was counterbalanced across participants. The contrast threshold was determined by averaging across the staircase endpoints, discarding the first four endpoints to account for initial learning. Compared to using the final tested value, averaging across endpoints is more representative of the participants' performance and is not as vulnerable to minor fluctuations in task performance.

## Results

### 2.1 Performance on the Reading, Phonological Awareness, and Language Measures

The means and standard deviations for all the reading, phonological awareness, and language measures for the poor readers and the non-impaired readers are in Table 1. Performance on all measures was compared across groups using MANOVA. The two groups were significantly different (F_(13,23)_ = 13.043, p<.001, η_p_^2^ = .881). Specifically, the non-impaired reader group significantly differed from the poor reader group on all measures, except for spatial relations. Moreover, the mean performance of the poor readers' scores on Word Identification, Word Attack, Spelling, TOWRE words, TOWRE non-words, Exception Words, and GORT fell below the minimum performance level of the non-impaired readers; this indicates that the majority of the poor readers were impaired on the reading and spelling measures. Based on the range of scores, the individuals in the poor readers group ranged from moderately to mildly impaired.

**Table 1:** Performance on the test battery by group.

	Non-Impaired Readers			Poor Readers				
	Mean (SD)	Min	Max	Mean (SD)	Min	Max	F	Sig.
Word Identification	110.00 (5.72)	99	122	93.44 (6.56)	82	106	67.07	<.001
Word Attack	106.10 (7.82)	96	118	86.38 (5.80)	75	94	71.55	<.001
Spelling	117.00 (10.29)	99	133	98.00 (6.87)	81	111	40.60	<.001
Verbal Comprehension	107.24 (8.96)	87	125	95.56 (7.75)	80	110	17.29	<.001
Spatial Relations	108.57 (8.08)	100	133	105.56 (9.53)	90	120	1.08	.306
Rapid Automatic Naming	107.29 (15.40)	73	135	94.81 (12.93)	64	121	6.82	.013
Auditory Working Memory	115.33 (14.06)	88	140	96.75 (8.57)	74	111	21.18	<.001
TOWRE words	108.88 (6.46)	96.5	114	87.50 (10.69)	64	107	57.05	<.001
TOWRE non-words	101.69 (6.33)	87.5	112	82.81 (5.30)	75.5	94	92.60	<.001
Nelson Denny Comp.	6.62 (1.66)	4	9	4.50 (1.59)	2	7	15.35	<.001
Exception Words	69.33 (0.80)	67	70	66.06 (2.05)	63	70	44.98	<.001
GORT Passage scores	13.95 (2.16)	9	16	8.19 (2.48)	2	13	56.98	<.001
Phoneme Elision	10.81 (1.72)	4	12	8.94 (2.43)	3	11	7.52	0.010

*Note*. Values in the table are based on standardized scores, except for Nelson Denny Comp. (stanines; max: 9), Exception Words (raw score; max: 70), GORT Passage Score (standard score; max for this age range: 16), and Phoneme Elision (standard score; max for this age range: 12).

### 2.2 Practice Trials: With and Without Noise

Two practice conditions preceded the experimental conditions. Both practice conditions had a small stimulus set size (i.e. ‘x’ always in the center of the stimulus region) and had thirty trials, but one condition was presented without noise and the other set of trials contained background noise. The groups did not significantly differ in terms of final step size (F_(2, 34)_ = 1.225, p = .306, η_p_^2^ = .067) nor number of m_shifts_ (F_(2, 34)_ = 0.417, p = .663, η_p_^2^ = .024) for the practice trials. This indicates that comparable stopping criteria were used for both groups.

The means and standard deviations for the practice and experimental conditions are shown in Table 2. Performance on the practice trials was compared between groups using a parametric independent t-test for the condition without noise and due to unequal variances in the trials with noise, the unequal variance t-test was used to compare performance on those trials. Performance significantly differed between the groups only on trials that contained external noise (t_29.64_ = −2.428, p = .021, d = 0.820), but not on trials without external noise (t_35_ = −1.631, p = .112, d = 0.536). The alpha was Bonferroni corrected to .025. Thus, poor readers performed worse on the letter detection task with a small stimulus set only when the trials contained noise. The absence of an advantage for good readers on the practice trials is inconsistent with the perceptual anchor hypothesis, as anchoring effects are posited to occur fairly quickly, that is, the non-impaired readers would have learned the task fast and performed better than the poor readers in this task [10], [28].

**Table 2:** Contrast threshold means and standard deviations for the practice and experimental conditions by group.

Condition	Group	Mean (SD)
No noise, small stimulus set (practice condition)	Non-Impaired Readers	0.182 (0.077)
	Poor Readers	0.222 (0.065)
Noise, small stimulus set (practice condition)	Non-Impaired Readers	0.519 (0.150)
	Poor Readers	0.609 (0.069)
No noise, small stimulus set (experimental condition)	Non-Impaired Readers	0.167 (0.050)
	Poor Readers	0.175 (0.049)
No noise, large stimulus set (experimental condition)	Non-Impaired Readers	0.255 (0.087)
	Poor Readers	0.287 (0.099)
Noise, small stimulus set (experimental condition)	Non-Impaired Readers	0.493 (0.102)
	Poor Readers	0.565 (0.142)
Noise, large stimulus set (experimental condition)	Non-Impaired Readers	0.668 (0.127)
	Poor Readers	0.756 (0.154)

*Note:* All values represent raw, non-standardized scores on the visual letter detection task.

### 2.3 Experimental Conditions: Main Effects and Interactions

**Figure 1:**
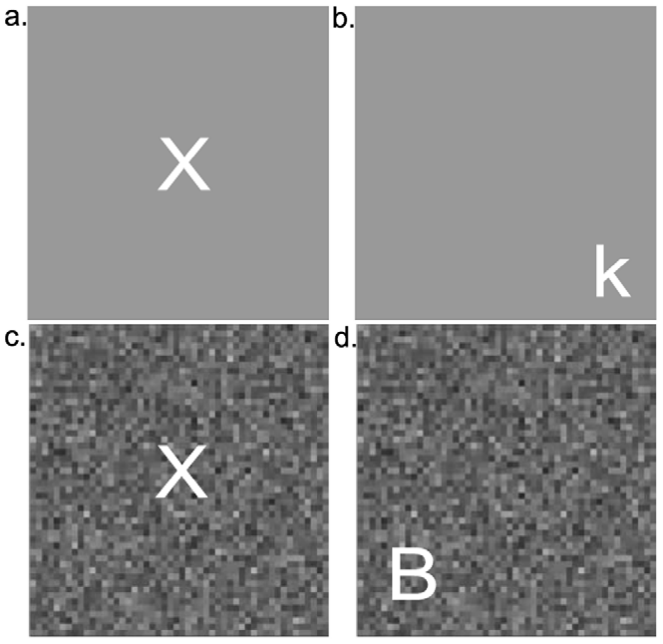
Examples of stimuli used in the four experimental conditions of the letter detection task. a.) Without noise, small stimulus set size; b.) Without noise, large stimulus set size; c.) Noise, small stimulus set size; d.) Noise, large stimulus set size.

**Figure 2:**
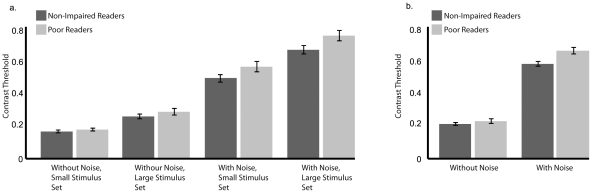
The mean contrast threshold values by group for a) each experimental condition and b) for the trials with and without noise. Error bars represent s.e.m.

To evaluate both the external noise exclusion and the perceptual anchoring hypotheses, the participants performed four experimental conditions that varied both stimulus set size as well as the presence of background noise (see Figure 1). The means and the standard error of the mean for the four experimental conditions are displayed in Figure 2. Each of the experimental conditions contained fifty trials and the order was counterbalanced across subjects. Like the practice trials, the groups did not significantly differ on the size of the final step (F_4, 32_ = 0.420, p = .793, η_p_^2^ = .050) nor the number of m_shifts_ (F_4, 32_ = 0.560, p = .694, η_p_^2^ = .065).

We conducted a 2×2×2 repeated measures ANOVA to evaluate the main effects of and interactions between external noise condition (with noise vs. without noise), stimulus set size (small vs. large), and reading group (non-impaired readers vs poor readers). There were significant main effects of external noise (F_1,35_ = 752.822, p<.001, η_p_^2^ = .956) and stimulus set size (F_1,35_ = 57.249, p<.001, η_p_^2^ = .621). As expected, performance was worse for both groups in the noise condition, and in the large stimulus set size condition. Moreover, there was a significant interaction between noise and set size (F_1,35_ = 5.265, p = .028, η_p_^2^ = .131). There was a significant effect of stimulus set size for the trials without noise (t_36_ = −8.386, p<.001, d = 1.333) and with noise (t_36_ = −5.342, p<.001, d = 1.349). Looking at the estimated marginal means, the difference in means for the large stimulus set vs small stimulus set was greater for noise (.712 vs .529) relative to the trials without noise (.271 vs .171). This indicates the trials with noise and a large stimulus set were more difficult for all of the participants.

In line with the external noise exclusion hypothesis, there was a significant interaction between reading group and noise condition (F_1,35_ = 4.205, p = .048, η_p_^2^ = .107). Conversely, there was no significant interaction between stimulus set size and reading group (F_1,35_ = 0.283, p = .598, η_p_^2^ = .008), which is inconsistent with the perceptual anchor hypothesis as it would predict that the poor readers would perform worse on trials with small stimulus sets. There was also no significant three-way interaction between reading group, stimulus set, and noise (F_1,35_ = 0.009, p = .927, η_p_^2^<.001).

### 2.4 Performance in Experimental Conditions With and Without Noise

To explore the significant interaction between noise and reading group, we performed two planned group comparisons using two parametric independent t-tests with the alpha Bonferroni corrected to .025. These tests revealed that the non-impaired readers and the poor readers significantly differed when the trials contained distracting background noise (t_35_ = −3.114, p = .004, d = 1.026), but not when the trials were free of noise (t_35_ = −0.939, p = .354, d = 0.311). The means and the standard error of mean for the conditions with and without noise are displayed in Figure 2. Thus, the poor readers performed significantly worse than the non-impaired readers in conditions that contained distracting background information, regardless of stimulus set size.

## Discussion

The poor readers performed significantly worse than the non-impaired readers on the letter detection task only in the high external noise conditions, regardless of stimulus set size. These results support the external noise exclusion hypothesis, which posits that poor readers have a general deficit in filtering out irrelevant information when attending to a signal [5]. This deficit theoretically results in the creation of representations of letters and sounds that contain irrelevant information in addition to the target. Contrary to the perceptual anchor hypothesis, there was no significant interaction between stimulus set size and group. Thus, the poor readers and non-impaired groups showed similar anchoring patterns for both small and large stimulus set sizes and consequentially, the poor readers did not have a particular problem taking advantage of repeated stimulus presentation in the letter detection task. Moreover, there was no evidence supporting the alternative hypothesis that the additive effects of noise and stimulus set size differed by group. We also analyzed the practice trial data, as anchoring effects might be posited to occur during these trials [10], [28]. However, the results were similar for practice and experimental trials (group by noise interactions but not group by stimulus set size interactions).

These results indicate that poor readers' ability to categorize and represent a letter, a skill necessary to learn the letter-to-sound correspondences in a language, is impaired only when external noise is present. This finding adds to previous studies, which show that dyslexic children and adults' perception of visual signals in the presence of external noise is significantly impaired relative to non-impaired readers [5], [9]. Combined with findings that dyslexic individuals have similar difficulties excluding background information in speech perception tasks [6]–[8], the external noise exclusion deficit appears to be a broader deficit that affects both the auditory and visual modalities.

The poor readers were significantly worse on all reading, phonological awareness, and language measures relative to the non-impaired readers, but performed as well as non-impaired readers on a measure of non-verbal intelligence, spatial relations. This pattern of results is consistent with the phonological core deficit theory in that poor reading was accompanied by impaired phonological processing, as measured by the rapid automatic naming, auditory working memory, and phoneme elision tasks. Additionally, the poor reader group was significantly worse on verbal comprehension compared to the non-impared group. Previous studies of external noise exclusion have found that oral language skills mediated the relationship between noise exclusion and reading scores [9]. Due to the small sample size in the groups in the current study, we were unable to directly examine the mediating role of oral language in the relationship between external noise exclusion and reading.

Although the present study is not the first failure to replicate the perceptual anchor hypothesis [29], the failure to show the perceptual anchor effect in the visual letter detection task adds to the debate as to why the anchoring deficit is found using some tasks with small stimulus sets, but not others, including: speech perception, rise time perception, intensity discrimination, and rapid naming [28], [30]. These inconsistent experimental findings call into question the overall explanatory power of the perceptual anchor hypothesis. It is possible that dyslexic individuals may exhibit the anchoring deficit in some conditions; however, the anchoring deficit may be caused by a broader perceptual impairment and thus, may be a secondary, rather than primary, impairment in dyslexia.

Although our results strongly support the external noise exclusion hypothesis, there are some limitations in generalizing the findings from this study. The members of the poor reader group ranged from moderately to mildly impaired, which could be why anchoring effects were not observed. However, an anchoring deficit has previously been observed with a sample of similarly impaired readers [14]. This suggests that the degree of impairment for the poor readers was likely not the reason why an anchoring effect was absent in this study.

The present study is also limited in that it does not address whether the external noise exclusion problem, rather than the anchoring problem, is present earlier in development. If young children at risk for developing dyslexia have an early difficulty in separating signal from noise, we hypothesize that this impairment would directly affect the efficiency of the neural network involved in representing letters, phonemes and their associations. For example, children in the initial stages of reading acquisition focus serially on single letters in order to link those letters to single phonemes [31]. Similar to Harm and Seidenberg's [32] computational model, we propose that the external noise exclusion deficit would lead to imprecise representations and difficulty matching incoming visual and auditory information to stored representations. Consequentially, letters and phonemes would not be represented in sufficient detail to support the development of phonological awareness and the learning of letter-sound correspondences, which would lead to reading problems. Therefore, a deficit in excluding distracting background information could be more strongly related to the emergence and development, rather than mastery, of phonological awareness.

Further studies should examine whether training children at-risk for dyslexia to perceive visual and auditory signals in noise is an effective way to reduce the prevalence or severity of reading and language impairments. Boets et al [8] found that speech perception in noise deficits are present in kindergartners who were later diagnosed with dyslexia. This study provides longitudinal evidence linking early noise exclusion deficits to later reading problems. Although no directional relationships were observed, these results raise a question as to whether early training in noise exclusion may lessen the severity of later reading problems.

In conclusion, our results indicate that an external noise exclusion deficit, but not a perceptual anchoring deficit, is present in undergraduate, poor readers. Further studies are needed to clarify the role of this deficit during reading acquisition and whether interventions can benefit reading development.
